# Surgical outcomes and cost analysis of a multi-specialty robotic-assisted surgery caseload in the Australian public health system

**DOI:** 10.1007/s11701-023-01643-6

**Published:** 2023-06-08

**Authors:** Daniel Steffens, Kate E. McBride, Nicholas Hirst, Michael J. Solomon, Teresa Anderson, Ruban Thanigasalam, Scott Leslie, Sascha Karunaratne, Paul G. Bannon

**Affiliations:** 1 RPA Institute of Academic Surgery (IAS), Royal Prince Alfred Hospital and University of Sydney, Missenden Road, PO Box M40, Sydney, NSW 2050 Australia; 2 Surgical Outcomes Research Centre (SOuRCe), Royal Prince Alfred Hospital, Sydney, NSW Australia; 3 Faculty of Medicine and Health, Central Clinical School, The University of Sydney, Sydney, NSW Australia; 4 The Baird Institute, Sydney, NSW Australia

**Keywords:** Robotic-Assisted Surgery, Benign Gynaecology, Colorectal, Urology, Surgical outcomes, Cost

## Abstract

This study aims to compare surgical outcomes and in-hospital cost between robotic-assisted surgery (RAS), laparoscopic and open approaches for benign gynaecology, colorectal and urological patients and to explore the association between cost and surgical complexity. This retrospective cohort study included consecutive patients undergoing RAS, laparoscopic or open surgery for benign gynaecology, colorectal or urological conditions between July 2018 and June 2021 at a major public hospital in Sydney. Patients’ characteristics, surgical outcomes and in-hospital cost variables were extracted from the hospital medical records using routinely collected diagnosis-related groups (DRG) codes. Comparison of the outcomes within each surgical discipline and according to surgical complexity were performed using non-parametric statistics. Of the 1,271 patients included, 756 underwent benign gynaecology (54 robotic, 652 laparoscopic, 50 open), 233 colorectal (49 robotic, 123 laparoscopic, 61 open) and 282 urological surgeries (184 robotic, 12 laparoscopic, 86 open). Patients undergoing minimally invasive surgery (robotic or laparoscopic) presented with a significantly shorter length of hospital stay when compared to open surgical approach (P < 0.001). Rates of postoperative morbidity were significantly lower in robotic colorectal and urological procedures when compared to laparoscopic and open approaches. The total in-hospital cost of robotic benign gynaecology, colorectal and urological surgeries were significantly higher than other surgical approaches, independent of the surgical complexity. RAS resulted in better surgical outcomes, especially when compared to open surgery in patients presenting with benign gynaecology, colorectal and urological diseases. However, the total cost of RAS was higher than laparoscopic and open surgical approaches.

**Supplementary Information:**

The online version contains supplementary material available at 10.1007/s11701-023-01643-6.

## Introduction

Robotic-assisted surgery (RAS) offers a minimally invasive approach for operations that have traditionally required a large open incision, and the expanding literature indicates that is a safe alternative with improved perioperative outcomes. The uptake of RAS has increased significantly over the past decade, with many common gynaecological, colorectal and urological procedures now taking advantage of the benefits afforded by robotic technology [1]. With the added benefits of RAS including stereoscopic, magnified vision, fully wristed articulation from endowristed instruments there has been a proportional decrease in not only open, but also conventional laparoscopic cases [2]. Despite the wide and rapid adoption of RAS, evidence demonstrating the superiority of RAS on key surgical outcomes when compared to laparoscopic or open approaches is mixed. Randomised controlled trials comparing RAS with conventional laparoscopic or open techniques have been undertaken in gynaecology, colorectal and urological surgery which have largely demonstrated equivalence in surgical outcomes [3–7]. One of the reasons for this may be that RAS technology is still evolving, and these outcomes are reflective of the steep learning curve of the surgical team [8]. However, recent systematic reviews of mostly non-randomised comparative studies have described some potential benefits of RAS on surgical outcomes across multiple specialties when compared to laparoscopic and open approaches [9–11]. While there are many research studies describing outcomes following RAS, the surgical outcomes based on case complexity levels and in-hospital cost of RAS compared to laparoscopic or open surgery is still under investigated, especially in the public sector [12, 13].

A recent publication detailed the implementation (including cost of the da Vinci Xi and sterilising equipment), maintenance and episode cost of RAS in public patients undergoing colorectal, gynaecology, cardiothoracic and urological procedures [14]. While this study provides important information on the implementation cost of RAS across multiple surgical specialties, it failed to compare RAS costs to conventional laparoscopic or open approaches. Thus, detailed comparison of RAS in-hospital costs would be of importance to health providers and policy makers.

The overall cost of RAS compared to other surgical approaches, specifically in urology, colorectal and gynaecology disciplines, is somewhat conflicting. While some studies report the overall cost of RAS to be significantly higher [15–18], others report it to be lower [19] or demonstrate no difference in costs [20]. The current limitations of these studies are that most lack details regarding cost variables and most do not take into account the complexity of the procedures. This is important when comparing the cost of procedures utilising new technology, as most surgeons tend to select simpler cases when using less familiar equipment. While the selection of surgical approaches are multi-factorial, this still represents the true cost of RAS to the health system [1]. Detailed information is important for hospitals that plan RAS within their surgical treatment options in the future and for patient decision-making.

Therefore, the primary aims of this study were to compare surgical outcomes and in-hospital costs between consecutive patients undergoing robotic, laparoscopic and open surgery for benign gynaecology, colorectal and urological diseases in the public hospital setting. The secondary aim was to investigate the impact of surgical complexity on in-hospital costs.

## Methods

### Study design and setting

This retrospective cohort study included all patients who underwent benign gynaecology, colorectal and urological surgeries from July 2018 and June 2021 at the Royal Prince Alfred Hospital, Sydney. It was one of the first public tertiary referral hospitals in Australia to purchase the da Vinci Xi robotic surgical system with the first urological case performed in October 2016, followed by colorectal and begin gynaecology in October and November 2017, respectively [21]. This study obtained Ethics approval and Governance authorisation from the Sydney Local Health District Human Research Ethics Committee (Royal Prince Alfred Hospital Zone, HREC reference number 2019/ETH13577). This manuscript followed the reporting recommendations from the STROBE statement [22].

### Participants

Consecutive adult patients (≥ 18 years old) undergoing robotic, laparoscopic or open benign gynaecology (i.e. hysterectomy and excision of endometriosis), colorectal (i.e. anterior rectal resection and right hemicolectomy), and urological surgeries (i.e. partial nephrectomy and radical prostatectomy) were reviewed. Patients were identified and grouped according to the Australian Refined Diagnosis Related Groups version 10.0 (AR-DRG V10.0) classification system [23]. All data was extracted by a senior member of the Sydney Local Health District Performance Unit.

### Patient characteristics and surgical outcomes

The following characteristics and surgical outcomes were extracted for all patients: Age (years), sex (male/female), country of birth (Australia/overseas), surgical emergency (elective/emergency), insurance status (public/private), length of intensive care unit stay (days), length of hospital stay (days), hospital re-admission within 28 days, and surgical complexity. Surgical complexity was defined according to the AR-DRG patient clinical complexity level code and grouped as minor (DRG C and D) or intermediate/major (DRG A and B) [23].

Overall number of postoperative complications and specific postoperative complications were recorded as gastrointestinal (i.e. anastomotic leak, intestinal injury, peritoneal injury, stomal complication), genitourinary (i.e. anastomotic leak, bladder injury, ureteric injury, uterine injury and ovarian injury), respiratory (i.e. pneumothorax, diaphragmatic injury), wound (i.e. wound infection, wound dehiscence), haemorrhage, thrombosis/embolism complications and in-hospital mortality.

### In-hospital cost

The in-hospital cost associated with each individual admitted patient was calculated from the annual inpatient fractions (iFRACs) [24]. This is estimated from the proportion of total hospital expenditure that related to the provision of care for admitted patients. The total in-hospital cost for each patient was calculated from the sum of following surgical cost variables: (i) staff (medical, nursing and allied health); (ii) critical care; (iii) emergency; (iv) diagnostics (imaging, pathology, pharmacology, prosthetics, specialist procedure suites); (v) operating room; (vi) ward; and (vii) other costs (hotel, non-clinical and on-costs) [14]. The complete description of the in-hospital cost variables is presented in the Supplementary Table 1. All in-hospital cost variables are reported in Australian Dollars (AUD).

### Statistical analysis

All data was collated and managed using an online platform (REDCap) [25], and the statistical calculations were conducted using SPSS version 28. Patient characteristics, surgical outcomes, and in-hospital cost data were presented as frequency (percentage) or median (interquartile range). Comparisons between categorical data were conducted using Chi-Squared Test or Fisher’s Exact Test (i.e. for cell count < 5). The Kruskal–Wallis one-way analysis of variance was used to compare continuous surgical outcomes and in-hospital cost data across robotic, laparoscopic and open surgical approaches. A subgroup analysis was conducted to compare in-hospital cost according to surgical complexity (minor versus intermediate and major complexity). For all analyses performed a p value < 0.05 was indicative of statistical significance and all tests were two-sided.

## Results

### Sample characteristics

Over the studied period, a total sample of 1,271 participants were included covering 287 (22.6%) robotic, 787 (61.9%) laparoscopic and 197 (15.5%) open. Of the total, 756 (59.5%) underwent benign gynaecological (robotic = 54, laparoscopic = 652, open = 50), 233 (18.3%) underwent colorectal (robotic = 49, laparoscopic = 123, open = 61) and 282 (22.2%) underwent a urological procedure (robotic = 184, laparoscopic = 12, open = 86).

The characteristics and surgical outcomes of the included sample are presented in Table 1. The length of hospital stay was shorter in patients undergoing minimally invasive surgery (robotics or laparoscopic), when compared to open surgery (all p values < 0.001). Most of the patients undergoing colorectal (77.6%) and urological (94.0%) RAS were of minor complexity when compared to laparoscopic (67.5% and 66.7%, respectively) and open (44.3% and 52.3%, respectively) (both p values < 0.001).

**Table 1:** Characteristics of the included sample (N = 1271)

Variables	Benign Gynaecology (N = 756)			Colorectal (N = 233)			Urology (N = 282)		
	Robotic (N = 54)	Laparoscopic (N = 652)	Open (N = 50)	Robotic (N = 49)	Laparoscopic (N = 123)	Open (N = 61)	Robotic (N = 184)	Laparoscopic (N = 12)	Open (N = 86)
Age, years	38.5 (31.0 to 42.0)**	38.0 (31.0 to 45.0)**	49.0 (44.8 to 51.3)**	66.0 (52.5 to 75.5)	66.0 (54.0 to 79.0)	66.0 (58.5 to 76.5)	65.0 (59.3 to 69.0)	67.0 (59.8 to 75.3)	64.0 (52.8 to 70.0)
Sex									
Female	54 (100.0)	652 (100.0)	49 (100.0)	21 (42.9)	48 (39.0)	31 (50.8)	7 (3.8)**	5 (41.7)**	17 (19.8)**
Male	–	–	–	28 (57.1)	75 (61.0)	30 (49.2)	177 (96.2)**	7 (58.3)**	69 (80.2)**
Country of birth									
Australia	30 (55.6)	349 (53.5)	22 (44.0)	26 (53.1)**	56 (45.5)**	45 (73.8)**	66 (35.9)	4 (33.3)	36 (41.9)
Overseas	24 (44.4)	302 (46.3)	28 (56.0)	23 (46.9)**	67 (54.5)**	16 (26.2)**	118 (64.1)	8 (66.7)	50 (58.1)
Elective procedure	54 (100.0)	636 (97.5)	48 (96.0)	48 (98.0)*	108 (87.8)*	48 (78.7)*	183 (99.5)	11 (91.7)	84 (97.7)
Public patient	34 (63.0)	452 (69.3)	33 (66.0)	37 (75.5)	106 (86.2)	47 (77.0)	162 (88.0)*	0 (0.0)*	35 (75.6)*
ICU Stay	0 (0.0)	0 (0.0)	0 (0.0)	7 (14.3)	10 (8.1)	12 (19.7)	2 (1.1)*	0 (0.0)*	9 (10.5)*
ICU Stay, hours	–	–	–	65.0 (20.0 to 122.0)	55.5 (23.0 to 131.5)	44.0 (22.3 to 83.3)	25.0 (23.0 to –)	–	25.0 (14.0 to 69.5)
Length of Stay, days	1.0 (1.0 to 2.3)**	1.0 (1.0 to 2.0)**	3.0 (3.0 to 4.0)**	7.0 (5.0 to 10.0)**	7.0 (5.0 to 11.0)**	10.0 (7.0 to 21.0)**	2.0 (2.0 to 3.0)**	6.0 (4.0 to 7.0)**	7.0 (5.0 to 10.3)**
Hospital readmission	1 (1.9)*	32 (4.9)*	7 (14.0)*	11 (22.4)	23 (18.7)	6 (9.8)	1 (0.5)*	2 (16.7)*	3 (3.5)*
Surgical complexity									
Minor	20 (37.0)	240 (36.8)	37 (74.0)	38 (77.6)**	83 (67.5)**	27 (44.3)**	173 (94.0)**	8 (66.7)**	45 (52.3)**
Intermediate/Major	34 (63.0)	412 (63.2)	13 (26.0)	11 (22.4)**	40 (32.5)**	34 (55.8)**	11 (6.0)**	4 (33.3)**	41 (27.7)**

Data presented as median (interquartile range) or frequency (percentage); *ICU* intensive care unit, *P < 0.05, **P < 0.001

The rate of postoperative in-hospital complications is presented in Table 2. Colorectal and urological robotic procedures presented significantly lower rates of overall postoperative complications. Rates of gastrointestinal, genitourinary, respiratory, wound, haemorrhage, thrombosis/ embolism and in-hospital mortality were similar across surgical approaches.

**Table 2:** Detailed postoperative in-hospital complications

Variables	Benign Gynaecology (N = 756)			Colorectal (N = 233)			Urology (N = 282)		
	Robotic (N = 54)	Laparoscopic (N = 652)	Open (N = 50)	Robotic (N = 49)	Laparoscopic (N = 123)	Open (N = 61)	Robotic (N = 184)	Laparoscopic (N = 12)	Open (N = 86)
Postoperative complications	4 (7.4)	24 (3.7)	5 (10.0)	3 (6.1)*	16 (13.0)*	16 (26.2)*	3 (1.6)**	1 (8.3)**	14 (16.3)**
Gastrointestinal	2 (3.7)	12 (1.8)	1 (2.0)	3 (6.1)	7 (5.7)	8 (13.1)	2 (1.1)^	–	1 (1.2)^
Genitourinary	–	5 (0.8)^	1 (2.0)^	–	–	1 (1.6)^	1 (0.5)^	–	9 (10.5)^
Respiratory	–	–	–	–	–	1 (1.6)^	–	1 (8.3)^	–
Wound	–	1 (0.2)^	1 (2.0)^	1 (2.0)	4 (3.3)	6 (9.8)	–	–	3 (3.5)^
Haemorrhage	2 (3.7)	6 (0.9)	2 (4.0)	1 (2.0)	5 (4.1)	2 (3.3)	–	–	1 (1.2)^
Thrombosis/ Embolism	–	–	–	–	–	1 (1.6)^	–	–	–
In-Hospital Mortality	–	–	–	1 (2.0)	1 (0.8)	2 (3.3)	–	–	–

Data presented as frequency (percentage); *P < 0.05, **P < 0.001; ^P value not able to be estimated

### In-hospital costs

The detailed in-hospital cost comparisons across surgical approaches are presented in Table 3. All cost variables investigated were significantly different within benign gynaecology, colorectal and urological surgical procedures. Staff and diagnostic costs were significantly higher in open surgeries, whereas operation theatre and total costs were significantly higher in the robotic surgeries.

**Table 3:** In-hospital cost comparisons between robotic and non-robotic procedures

Cost variables	Benign Gynaecology (N = 756)			Colorectal (N = 233)			Urology (N = 282)		
	Robotic (N = 54)	Laparoscopic (N = 652)	Open (N = 50)	Robotic (N = 49)	Laparoscopic (N = 123)	Open (N = 61)	Robotic (N = 184)	Laparoscopic (N = 12)	Open (N = 86)
Staff	820.0 (583.3 to 1588.5)**	720.5 (400.5 to 1201.0)**	1931.0 (1474.0 to 2390.8)**	4860.0 (3569.0 to 6290. 5)**	4423.0 (3232.0 to 7621.0)**	7688.0 (4978.0 to 15,113.0)**	1989.5 (1735.5 to 2678.3)**	4696.5 (3442.0 to 6342.3)**	5572.5 (4081.5 to 7188.8)**
Critical Care	–	–	–	5717.0 (1710.0 to 9005.0)	8697.0 (3257.8 to 13,863.8)	4030.5 (2004.3 to 7664.3)	2910.5 (1275.8 to 9854.5)	–	3509.0(2321.5 to 5987.5)
Diagnostic	225.0 (20.5 to 530.0)*	180.0 (19.0 to 309.5)*	269.0 (83.3 to 382.8)*	1593.0 (971.0 to 2095.0)*	1,545.0 (1003.0 to 2249.0)*	2060.0 (940.0 to 4716.0)*	890.0(410.5 to 1402.0)**	1590.0 (716.5 to 2061.8)**	2061.5(1160.3 to 4037.5)**
Operation Theatre	12,407.0 (10,545.8 to 15,930.8)**	3163.5(2363.8 to 4258.8)**	3954.5 (3407.3 to 4927.5)**	18,842.0 (1670 5.5 to 21535.5)	7436.0 (5121.0 to 9974.0)**	9246.0 (6518.5 to 12,044.0)**	17,667.5(15,503.0 to 19,374.5)**	7401.0(6378.5 to 9995.8)**	9248.0 (6910.0 to 11,551.0)**
Ward	168.5(125.8 to 273.5)**	155.0 (107.3 to 239.8)**	349.0 (311.5 to 455.5)**	966.0 (717.0 to 1281.5)**	802.0 (579.0 to 1473.0)**	1429.0 (932.0 to 2689.5)**	318.0 (285.3 to 423.8)**	734.0 (530.3 to 992.5)**	880.0 (664.5 to 1227.3)**
Other	2203.0 (1724.0 to 2522.5)**	1022.5(745.3 to 1416.8)**	1817.0 (1574.0 to 2095.3)**	5272.0 (4323.0 to 6584.0)**	3892.0 (2988.0 to 5680.0)**	5932.0 (4177.0 to 10,602.5)**	3195.5 (2903.3 to 3792.0)**	3600.5 (3080.5 to 4323.5)**	4366.0 (3439.8 to 6076.0)**
Total Cost	16,457.5 (13,154.5 to 19,935.5)**	5280.0 (3898.5 to 7583.5)**	8571.0 (7478.8 to 10,332.0)**	32,496.0 (27,66 6.0 to 38778.0)**	19,430.0 (14,070.0 to 26,407.0)**	28,328.0 (19,652.5 to 49,233.5)**	24,083.0 (21,749.8 to 26,889.0)*	17,791.0 (16,554.0 to 22,104.0)*	21,895.0 (17,744.0 to 32,263.8)*

Data presented as median (interquartile range), *P < 0.05; **P < 0.001

The in-hospital cost of robotic, laparoscopic and open approaches according to surgical complexity are presented in Table 4. Total cost of robotic procedures were significantly higher across all specialties, independent of the surgical complexity, apart from urological intermediate/major complexity procedures, where no difference in cost was observed across the surgical approaches.

**Table 4:** In-hospital cost comparisons between robotic and laparoscopic procedures according to surgical complexity

Benign Gynaecology	Minor complexity (N = 297)			Intermediate/major complexity (N = 166)		
	Robotic (N = 20)	Laparoscopic (N = 240)	Open (N = 37)	Robotic (N = 14)	Laparoscopic (N = 139)	Open (N = 13)
Staff	1236.5 (800.5 to 1929.0)**	1180.5 (801.3 to 1529.5)**	1792.0 (1412.5 to 2161.0)**	863.0 (713.5 to 2122.5)**	723.0 (515.0 to 1240.0)**	2435.0 (1907.0 to 3148.5)**
Critical Care	–	–	–	–	–	–
Diagnostic	225.0 (57.5 to 533.0)**	216.0 (37.5 to 311.8)**	268.0 (66.5 to 340.5)**	297.0 (19.0 to 550.5)*	174.0 (18.0 to 320.0)*	394.0 (129.0 to 747.5)*
Operation Theatre	12,989.5 (10,879.5 to 14,824.5)**	3658.8 (3013.8 to 4643.8)**	3910.0 (3401.5 to 4939.0)**	14,879.5 (10,983.3 to 16,714.3)**	3484.0 (677.0 to 5049.0)**	4276.0 (3339.0 to 4819.0)**
Ward	227.0 (146.3 to 308.3)**	233.0 (152.3 to 271.0)**	341.0 (274.5 to 385.0)**	181.0 (149.5 to 428.0)**	158.0 (119.0 to 249.0)**	489.0 (329.0 to 572.0)**
Other	2217.0 (1990.0 to 2484.8)**	1324.0 (1016.8 to 1660.3)**	1764.0 (1540.5 to 2018.5)**	2351.0 (1825.5 to 3212.3)**	1158.0 (837.0 to 1513.0)**	2182.0 (1632.5 to 2912.0)**
Total Cost	17,115.0 (14,538.3 to 19,286.5)**	6658.0 (5228.5 to 8557.5)**	8165.0 (7365.0 to 9483.5)**	19,694.5 (13,521.3 to 23,200.3)**	5784.0 (4209.0 to 8043.0)**	10,044.0 (8023.0 to 12,494.5)**

Colorectal	Minor complexity (N = 148)			Intermediate/major complexity (N = 85)		
	Robotic (N = 38)	Laparoscopic (N = 83)	Open (N = 27)	Robotic (N = 11)	Laparoscopic (N = 40)	Open (N = 34)
Staff	4545.5 (3179.0 to 5358.3)*	3839.0 (2824.0 to 4789.0)*	5197.0 (4075.0 to 7802.0)*	6607.0 (4845.0 to 20,026.0)	7929.3 (4429.3 to 12,215.0)	9967.5 (6299.0 to 19,209.0)
Critical Care	1710.0 (732.0 to 1710.0)	3676.0 (2003.0 to –)	1975.0 (1607.0 to 5753.0)	7894.0 (5486.0 to 40,178.8)	8749.0 (8143.0 to 13,339.0)	4310.0 (3309.5 to 10,560.5)
Diagnostic	1492.0 (855.8 to 1983.0)	1372.0 (852.0 to 1801.0)	1764.0 (677.0 to 2671.0)	2120.0 (1802.0 to 6083.0)	2441.0 (1378.0 to 4708.8)	2989.0 (1592.0 to 7431.5)
Operation Theatre	19,080.5 (16,586.5 to 21,111.5)**	7223.0 (4960.0 to 9666.0)**	9592.0 (7000.0 to 12,034.0)**	18,452.0 (16,876.0 to 25,988.0)**	8000.5 (5190.8 to 11,957.8)**	9151.0 (6362.0 to 13,416.3)**
Ward	934.5 (591.0 to 1097.3)*	700.0 (536.0 to 871.0)*	948.0 (693.0 to 1457.0)*	1352.0 (991.0 to 4375.0)	1601.0 (868.5 to 2362.3)	1889.0 (1096.0 to 3655.8)
Other	4819.5 (4151.0 to 6072.8)**	3414.0 (2596.0 to 4455.0)**	5103.0 (3470.0 to 6840.0)**	6973.0 (4634.0 to 13,480.0)	6049.5 (3810.8 to 8586.3)	7963.5 (4984.5 to 12,886.3)
Total Cost	30,306.0 (27,233.0 to 36,101.8)**	16,495.0 (12,981.0 to 22,304.0)**	25,693.0 (16,278.0 to 29,972.0)**	42,305.0 (30,310.0 to 64,899.0)*	27,821.5 (18,866.8 to 39,592.8)*	35,493.0 (24,316.0 to 57,302.8)*

Urological	Minor complexity (N = 226)			Intermediate/major complexity (N = 56)		
	Robotic (N = 173)	Laparoscopic (N = 8)	Open (N = 45)	Robotic (N = 11)	Laparoscopic (N = 4)	Open (N = 41)
Staff	1976.0 (1737.0 to 2640.0)**	3750 (3291.5 to 5260.0)**	4601.0 (3529.5 to 5858.0)**	2534.0 (1692.0 to 4914.0)*	6237.5 (5441.5 to 6752.3)*	6562.0 (5246.0 to 12,155.0)*
Critical Care	2039.0 (840.0 to –)	–	1913.5 (1485.0 to –)	7321.5 (2583.0 to –)	–	4586.0 (3288.0 to 6699.0)
Diagnostic	891.0 (412.0 to 1389.0)**	1350.0 (716.0 to 2061.8)**	1563.0 (1138.5 to 2410.0)**	815.0 (405.0 to 2500.0)*	1627.5 (646.5 to 2436.5)*	3317.0 (1240.5 to 4866.0)*
Operation Theatre	17,685.0 (15,527.0 to 19,373.0)**	7382.0 (6378.0 to 9540.8)**	8977.0 (6737.0 to 10,310.5)**	17,091.0 (13,284.0 to 22,039.0)**	8543.5 (6081.8 to 15,580.3)**	9455.0 (7347.0 to 12,508.5)**
Ward	317.0 (284.0 to 420.5)**	593.0 (497.8 to 949.8)**	785.0 (601.0 to 922.0)**	386.0 (291.0 to 704.0)**	876.0 (819.5 to 992.5)**	1049.0 (811.0 to 1972.5)**
Other	3192.0 (2898.0 to 3747.0)**	3190.0 (3038.8 to 4097.8)**	3767.0 (3230.0 to 4805.0)**	3477.0 (2902.0 to 4549.0)*	4076.5 (3812.3 to 5873.8)*	5384.0 (4090.5 to 9306.5)*
Total Cost	24,054.0 (21,813.5 to 26,731.5)**	17,490.0 (15,825.8 to 19,092.3)**	20,674.0 (15,941.5 to 24,327.0)**	24,491.0 (21,062.0 to 30,824.0)	20,498.0 (17,687.0 to 31,613.0)	28,181.0 (20,507.0 to 45,196.0)

Data presented as median (interquartile range); *P < 0.05, **P < 0.001

## Discussion

Most of the differences in surgical outcomes and costs were observed between RAS and open approaches. This included a reduction in hospital stay of two days in benign gynaecology, three days in colorectal and five days in urological procedures. In addition, for colorectal (6.1% versus 26.2%) and urological (1.6% versus 16.3%) RAS, the rate of postoperative morbidity was significantly lower than the open approach. However, despite the significant benefits observed on surgical outcomes, the in-hospital cost of RAS was significantly higher than other surgical approaches. Overall, the surgical outcomes from our multi-specialty RAS caseload in the Australian public health system are encouraging. When comparing surgical outcomes across different surgical approaches, it is important to note that in some variables, for example length of hospital stay between RAS and laparoscopic benign gynaecology, it may not be possible to demonstrate a significant difference between surgical approaches, as both already result in a small length of hospital stay (i.e., median = 1.0 day). Whereas in other surgical procedures, including colorectal and urology, where the length of hospital stay for open procedures tend to be for longer periods, there is an opportunity for new technology to enhance recovery and reduce length of stay.

Only a limited number of studies have reported RAS surgical outcomes and in-hospital costs, compared to other surgical approaches, in the public health sector. A recent Canadian study reported outcomes following the first 104 patients that underwent robotic-assisted prostatectomy compared to 121 laparoscopic prostatectomies. In this series, the length of hospital stay was shorter in the robotic group, but the rate of postoperative complication was comparable across the groups [26]. One of the reasons for the improved RAS surgical outcomes in our study may be associated with the selection of the surgical approach being based on surgeons’ preference. A recent study described the patterns of care for radical prostatectomy in the public health system, where RAS has become the dominant surgical approach to treat patients presenting with prostate cancer [27]. The results of our study will further support the decision-making process for surgeons in selecting the best surgical approach with and for their patients.

This study compared surgical outcomes and in-hospital cost of consecutive patients undergoing benign gynaecology, colorectal and urological surgical procedures across robotic, laparoscopic and open approaches. This data was collected from a comprehensive, multi-specialty, publically funded RAS program with a unique research framework, whereby every patient being operated on robotically is enrolled in a research project. This approach was initially selected so use of the robotic surgical system (da Vinci Xi) could be safely and appropriately implemented within the public hospital setting, and has evolved to ensure ongoing contribution to the evidence surrounding use of the technology [21, 28]. Thus, the data reported in this manuscript is from almost 24 months following the initial implementation of RAS at our hospital.

At our centre, the most common surgical approach varied across surgical specialty. For instance, benign gynaecology and colorectal cases were predominantly performed laparoscopically, whilst urological cases were predominantly performed with RAS. The present study demonstrated that RAS improved surgical outcomes for all three specialties, when compared to both laparoscopic and open approaches.

We have demonstrated that in robotic colorectal (77.6%) and urological (94.0%) cases, most of the patients were of minor surgical complexity. This has also been evidenced in other publications, which supports the selection of lower surgical complexity patients in order to maintain major postoperative complications at a minimum, while the surgical team upskill [29]. In our study, intermediate and major complexity cases performed via RAS seems to have a lower postoperative complication rate, especially when compared to open approach (e.g., Benign gynaecology = 11.8% vs 38.5%; Colorectal = 18.2% vs 41.2%; Urology = 18.2% vs 24.4%). Although, due to the nature of our study, this needs to be further investigated in future study with appropriate methodological design.

The detailed cost of RAS at our institution has been extensively described in our previous publication, with the implementation, maintenance, consumables and operating theatre as the main drivers of total cost [14]. When compared to laparoscopic and open approaches, theatre costs for the robotic approach was two to three times higher and the main factor contributing to the difference in total cost across surgical approaches. Previous studies have demonstrated a strong relationship between RAS in-hospital costs and surgical volume [30]. Where the cost of RAS could contribute to > 15% in low volume centres (e.g. < 4 RAS cases per week). Additionally, another factor associated with operating theatre costs is the experience of the surgical team. Under these conditions, operative times have been observed to decrease after the team learning curve period, and is reflected by lower operative theatre costs [31]. Thus, after RAS is considered safe and the team has overcome the learning curve, it is imperative that institutions seek to maximise the use of RAS to reduce costs associated with this surgical approach [32–34]. Although RAS admission costs are higher when compared to other surgical approaches, significant benefits are observed in shorter hospital stay and reduced postoperative complication rates. In addition, other potential benefits that are not captured in the current study are the reduction of postoperative pain, quality of life outcomes, and other functional outcomes including continence, pain form endometriosis, and bowel function.

Some of the strengths of this study are the comparison of surgical outcomes and in-hospital cost across multi-specialty RAS with laparoscopic and open approaches in a major referral public hospital. In addition, the detailed cost analysis presented in our study will help institutions to make important decisions on the implementation of new RAS programs. Some of the limitations of our study are that our data is from a single tertiary referral hospital, including a retrospective design focusing on short term in-hospital outcome measures, and the small sample size within some of the surgical approaches. In addition, decision of surgical approach was based on individual surgeons. Thus, caution should be taken when interpreting and generalising these results.

## Conclusion

The implementation of RAS in the public system demonstrated encouraging surgical outcomes when compared to more traditional surgical approaches such as laparoscopic and open surgery in patients undergoing surgery for benign gynaecology, colorectal and urological diseases. Despite this, the cost of RAS was higher than laparoscopic and open surgical approaches. The increase in RAS uptake over time and the introduction of other robotic surgery competitors to the market are likely to reduce the cost of RAS in the future.

## Supplementary Information

Below is the link to the electronic supplementary material.

Supplementary file1 (DOCX 14 KB)

## Data Availability

Data is available upon reasonable request.
