# Supplementary material for: Surgical outcomes and cost analysis of a multi-specialty robotic-assisted surgery caseload in the Australian public health system
Source: J Robot Surg. 2023 Jun 8;17(5):2237–45. doi: 10.1007/s11701-023-01643-6 (PMC10492768; doi:10.1007/s11701-023-01643-6)
Supplement: Supplementary file 1 — Supplementary file1 (DOCX 14 KB) [file 11701_2023_1643_MOESM1_ESM.docx]

| **Supplementary Table 1.** Detailed information of in-hospital cost variables | |
| --- | --- |
| **In-hospital cost variables** | **Description** |
| **Staff** | Medical: Cost of all Medical Salary and Wages and VMO Payments in Clinical Service or Ward cost centres.  Nursing: Cost of all Nursing Salary and Wages in Clinical Service or Ward cost centres.  Allied Health: Cost of all Goods & Services and Salary and Wages for Allied Health Cost centres. Including cost of Allied Health Salary and Wages costs occurred in Clinical Services Cost centres. |
| **Critical Care** | Cost of all Goods & Services, Salary and Wages and VMO Payments for Critical Care cost centres including ICU, HDU, CTICU, PSICU, NICU, PICU and CCU. |
| **Diagnostic** | Pathology: Cost of all Blood and Pathology Goods & Service cost centre.  Imaging: Cost of all Imaging Goods & Services.  Specialist procedure suites: Cost of all Goods & Services, Salary and Wages and VMO Payments for Specialist Procedures Suites.  Pharmacology: Cost of all Pharmacy Goods & Service cost.  Prosthetics: Cost of all Prosthesis costs in all cost centres. |
| **Operating Theatre** | Cost of all Goods & Services, Salary and Wages and VMO Payments for Operating Theatre cost centres. |
| **Ward** | Average cost of all Goods & Services for Clinical Service or Ward cost centres. |
| **Other costs** | Hotel: Cost of all food and domestic services.  Non-clinical: Amount of non-clinical costs including hotel and administrative costs, non-clinical salaries and wages.  On costs: Amount of on costs. These costs include Superannuation and Workers Compensation premium payments.  Excluded: The average amount of costs that are excluded from the NSW State price including all Depreciation, Annual Leave and LSL actuarial adjustment, Interest  Patient transport: Average cost of all Patient Transport costs in all cost centres  Cost of all Goods & Services for Clinical Service or Ward cost centres. |
| **Total in-hospital cost** | Sum of staff, critical care, diagnostic, operating theatre, ward and other costs. |
